# Dynamics of airway response in lung microsections: a tool for studying airway-extra cellular matrix interactions

**DOI:** 10.1186/s12929-016-0263-2

**Published:** 2016-05-12

**Authors:** Mohammad Afzal Khan

**Affiliations:** Department of Comparative Medicine, King Faisal Specialist Hospital and Research Centre, MBC 03, P.O. Box 3354, Riyadh, 11211 Kingdom of Saudi Arabiana

**Keywords:** Velocity of airway narrowing, ASM hyperresponsiveness, Airway kinetics, Lung microsections, Video micrometry

## Abstract

The biological configuration of extracellular matrix (ECM) plays a key role in how mechanical interactions of the airway with its parenchymal attachments affect the dynamics of airway responses in different pulmonary disorders including asthma, emphysema and chronic bronchitis. It is now recognized that mechanical interactions between airway tissue and ECM play a key regulatory role on airway physiology and kinetics that can lead to the reorganization and remodeling of airway connective tissue. A connective tissue is composed of airway smooth muscle cells (ASM) and the ECM, which includes variety of glycoproteins and therefore the extent of interactions between ECM and ASM affects airway dynamics during exacerbations of major pulmonary disorders. Measurement of the velocity and magnitude of airway closure or opening provide important insights into the functions of the airway contractile apparatus and the interactions with its surrounding connective tissues. This review highlights suitability of lung microsection technique in studying measurements of airway dynamics (narrowing/opening) and associated structural distortions in airway compartments.

## Background

Pulmonary disorders including asthma, emphysema and chronic bronchitis are characterized by the extensive airway tissue remodeling that perturbs the architecture of ASM and ECM interactions in lung [24, 43]. These changes are believed to be largely responsible for the various physiological outcomes and thus severity of disease [2, 7]. Most of the airway structural deformities are due to loss of ECM and airway wall composition as reported in asthma, which is also characterized by the thickening of the airway wall (including both increased ASM and connective tissue) and ASM hyperresponsiveness [7, 37, 64]. Emphysema, on the other hand, are characterized in part by airway wall destruction, sometimes accompanied by a degree of airway hyperresponsiveness [56, 62]. The physiological features of parenchymal destructions include loss of elastic recoil leading to airflow obstruction with resting and dynamic hyperinflation [54, 70]. More specifically, Chronic Obstructive Pulmonary Diseases (COPDs), which include emphysema, fibrosis and bronchitis are also associated with a greater deficit in pulmonary functions than that seen in asthma, yet relatively little is known about its underlying pathophysiological mechanisms (in contrast to the vast literature available for asthma) [27, 35, 62]. This is in part due to the fact that the functional/structural modifications seen in COPDs are predominantly found in the small airways [3, 14], which are not easily studied using conventional scientific approaches such as organ baths and force transducers [60] but these parameters are easily examined using the lung microsection approach [4, 38, 39, 46, 50].

## Advantages and limitations

Lung microsections represent an *ex-vivo* replacement to in-vivo respiratory functional measurements of airway bronchoconstriction [17, 71]. Furthermore, lung microsections have been applied successfully to investigate molecular mediators involved in airway response, to study Ca^+2^ signaling in ASMs [9, 11, 71] and immune reactions of biologically active agents [31]. The viability of lung microsections can be examined to control local respiratory responses induced after exposure of lung microsections to gradients of various neurotransmitters. This advantage helps to examine multiple substances in one experimental lung. With an increasing call to regulate the number of experimental animals used in research and to further minimize the animal distress, lung microsections represent a promising technique for pharmacological and toxicological evaluations. In contrast to other in-vitro models, like isolated airways [30, 48, 59, 65], tracheal, or bronchial rings [68], where contraction is either isotonic or isometric, in lung microsections parenchymal tethering resulted in more auxotonic contractions like in-vivo conditions. In addition, the relative contributions of the contractile and non-contractile components of the airways are very difficult to resolve using in vivo approaches, whereas the use of cellular preparations (i.e., cultured or enzymatically dissociated cells) provides little or no information about the interactions between the two [20]. Even the slicing procedure means some stress for the tissue, which is the same in all in-vitro preparation [49]. Lung microsections approach is very productive in measuring small airway functional and structural perturbations seen in chronic obstructive diseases, which are not easily studied using conventional techniques like organ bath and force transducers [16, 39].

Lung microsections approach has been adopted successfully in the mouse model of emphysema, which highlights the role of the ECM in airway contraction challenged to various neurotransmitters [16, 39]. In addition, the preclinical significance of this technique has been reported in understanding the magnitudes and velocities of airway narrowing in a murine model of asthma (allergen-induced airway hyperresponsiveness) and in murine model of COPDs [38, 39], which show different pattern of ASM-ECM tethering. However, decreased tethering (e.g., in emphysema) should lead to faster contractions and slower relaxations, whereas the opposite would be seen under conditions in which tethering forces are increased (e.g., in fibrosis) [13, 67]. Under parallel physiological in-vitro conditions, mouse lung microsections incubated overnight with proteolytic enzymes elastase or collagenase showed significant loss of tethering forces [38, 39]. In addition, during an airway response, airway constriction and velocity of narrowing is governed by the elastic load on the airway wall [22, 51], which is controlled through the lung parenchyma (via the alveolar attachments to the airway wall), the airway wall itself, and the internal resistance to shortening of the smooth muscle cell [2, 41, 47, 52]. In preclinical studies of COPD as well as an in-vitro murine lung microsection technique, which allows to examine the interactions between the small airways (down to 10 microns) and its surrounding parenchyma; these functional studies are also powerfully complemented by histological studies done on the very same slices from which the functional data were obtained [38, 39]. In addition, these lung microsections approach in murine model reproduces many of the features of asthma and COPDs and allows one to distinguish the effects on airway dynamics of parenchymal destruction *versus* fibrosis and other tissue remodeling events [16, 39]. This technique is technically highly efficient, and able to investigate precisely the mechanisms underlying structure/functional abnormalities in different samples collected from preclinical and clinical samples as well as to provide rapid assessment of the potential benefit of testing novel therapeutic interventions for the treatment of these diseases [38, 39].

One of the great advantage of the lung microsections is that up to 30–50 slices can be prepared from one mouse lung and much more from other species (rat, guinea pig, primate, or human), which offers the possibility to receive significant results using fewer animals where each of it is its own internal control [39]. The video imaging of lung microsections as they contract and relax in response to perturbations provides the optimal compromise between these two extremes: the *in-situ* organization of the airway wall and the contractility of the ASM cells are maintained, the interactions between the ASM and ECM can be observed directly, the relative contributions of the small and large airways can be compared, and histological examinations can be performed immediately afterwards on the very same tissue in which functional responses were obtained [5, 8–10, 46].

The limitations of lung microsection technique compared to in-vivo methods is that migration of cells from the lung into the blood or lymph nodes and trafficking of cells from blood into the lungs during immune/inflammatory responses cannot be assessed in this model because of the lack of definite circulatory system. However, all cell types relevant to induction of immune responses like dendritic cells, macrophages, or mast cells are present, and the cells remain in their normal architectural relationship and interact with each other and with other resident cells relevant for bronchoconstriction like smooth muscle cells or nerves.

### Airway-connective tissue perturbations in pulmonary diseases

Airways are highly dynamic and can actively constrict almost-to-total occlusion when smooth muscle is hyper-stimulated as seen in asthmatics and emphysema patients [46, 55]. The airway wall comprises a layer of ASM embedded within a delicate mesh of connective tissue matrix [2, 19], which modulates the movement (opening and closing) of airway during a normal and aberrant airway response and limits the airflow [45]. The structural integrity and stiffness of the airway wall are important determinants of airway lumen area during dynamic collapse in a forced expiration or when airway smooth muscle is constricted [2, 66]. In addition, structural properties of an airway impacts its contractile response and stiffness of the airway will influence and possibly control the extent to which an airway narrows owing to smooth muscle contraction [38, 39, 69]. Shortening of the ASM leads to stretching of the connective tissue matrix — composed of proteins such as collagen, elastin, fibronectin, etc. — providing in part to a recoil force, this opens up the airways following ASM relaxation [1].

Studies of the airways in healthy and in disease condition often use indices of the degree of ASM contraction: *e.g*., measures of airflow resistance in patients (forced expiratory volume in 1 second, FEV_1_) or whole animals, airway narrowing in lung slices, or grams tension in isolated airway smooth muscle strips [38]. It is assumed that measurement of the magnitude of airway contraction may provide information about the ASM lining the airways but it may not be informative about the passive non-contractile components of the airway wall [2, 38, 39]. Several observations pertaining to COPDs point to the importance of examining the airway smooth muscle and its interaction with the surrounding parenchyma (tethering), including an increased smooth muscle mass, and the beneficial effects of anti-cholinergic and β-agonists in the treatment of COPDs [6, 34, 62]. However, it is not known whether the inherent responsiveness of the small airways is altered (which would explain why the airways are relatively refractory to bronchodilator therapies), if there are changes in cholinergic regulation and signaling, whether the decreased pulmonary function (reflected in the decreased FEV_1_) is due to increased initial narrowing [15, 28, 56]. There are also questions pertaining to the pulmonary vasculature, including whether the pulmonary hypertension associated with COPDs is due to a change in smooth muscle responsiveness *per se*, or whether it is secondary to airway narrowing and the consequent hypoxia (via the classical hypoxic pulmonary vasoconstrictor response).

COPDs exerts a tremendous burden on society, yet relatively little is known about its underlying pathophysiological mechanisms. [42, 53] The current understanding of this disease suggests that the loss of lung parenchyma results in decreased lung function through direct and indirect mechanisms. [26] Directly, loss of parenchyma results in increased lung compliance, while the increased size of terminal air spaces would impair intra-alveolar ventilation leading to hypoxia [26, 54]. Indirectly, loss of elastic tissue would reduce tethering forces around the airway, resulting in a greater propensity for the airway to narrow [26, 55]. In combination, these functional abnormalities result in impaired gas exchange with significant systemic manifestations [72]. Several preclinical and clinical observations has pointed to the importance of the ASM and its interaction with the surrounding parenchyma (tethering) and their role in severity of disease [18, 40, 46, 50]. As reported in most of the clinical conditions, COPD is accompanied by changes in smooth muscle responsiveness, increased fibrosis and destruction of lung parenchyma with loss of elastic tethering forces [6, 29, 56, 62], but all of these changes are easily quantified using the thin lung microsection approach [9, 16, 39, 46, 50]. It has been reported in mouse models of COPDs and emphysema that video micrometry of lung microsection reproduces many of the features of COPDs and allow one to distinguish the effects on airway dynamics of parenchymal destruction *versus* fibrosis and other tissue remodeling events [39, 50]. Several observations pertaining to COPDs point to the importance of examining the ASM and its interaction with the ECM, and the beneficial effects of anti-cholinergic and agonists in the treatment of COPDs [9, 40]. There are also questions pertaining to the pulmonary vasculature, including whether the pulmonary hypertension associated with COPDs is due to a change in smooth muscle responsiveness or it is secondary to airway narrowing and the consequent hypoxia and these questions can easily be answered and actualized using lung microsections approach.

### Airway response to bronchocontrictors

Lung microsections examine dynamics of airway response (contraction/relaxation) and the interaction between the ASM with its surrounding connective tissue matrix, which play a fundamental role in determining disease severity [16, 39, 46]. In particular, increased connective tissue would both reduce the magnitude of airway narrowing in response to a standard excitatory stimulus/ bronchoconstrictor stimuli as well as the velocity at which that bronchoconstriction developed; on the other hand, the velocity of relaxation would be increased with the corresponding increased tethering force on the airways [38, 39]. Destruction of the airway wall or surrounding parenchyma, on the other hand, would manifest as increased magnitudes and velocity of contraction, with decreased velocity of relaxation, which provide clear understanding of airway-connective tissue interactions, and normal and sensitized conditions [2, 16, 38, 39]. Mathematically, whole process of airway-connective tissue interaction can be easily examined through speed of airway movement during opening and closing and the peak velocity of ASM shortening can be calculated using a simple mathematical algorithm, which numerically differentiates the values of area with respect to time [46, 61]. As reported earlier, velocity varies in two phases of airway responses, which each represents velocity of contraction (Vcon) and velocity of relaxation (Vrel) during one cycle of airway movement [38]. It was found in higher magnitude during phase of airway contraction with respect to velocity during phase of airway relaxation. The two velocities differ only because of requirement of energy; therefore, contraction phase is regarded as active process while relaxation process is slow and passive process [38, 61]. Nonetheless, velocity also affected in both in-vitro and in-vivo protease challenged lung microsections [39]. Disruption of ECM directly hampers contraction, relaxation velocity and consequently magnitude of airway contraction, which is very common phenomenon in major respiratory conditions [2, 9, 61]. All in all, velocity of airway responses reflect how perfect attachments /tethering around the airway network is. Lung microsections proved to be highly robust in understanding the velocity of the bronchoconstriction responses vary with airway size: in fact, velocities of changes in airway diameter to vary substantially or in a systematic manner between the smaller and larger airways [38]. The technique is therefore ideal for investigating the impact of tethering forces on airway mechanical responses: it is understood that such interactions are obviated in tracheal strips and bronchial rings (which lack the parenchyma) [21, 32]. The lung tissue is a viscoelastic material, and, viscoelastic materials display hysteresis. In some major clinical conditions, the elastin fibers are degraded (emphysema): as such, the airway wall does not recoil so strongly [2, 38, 46, 61]. On the other hand, pulmonary fibrosis with aging or in disease would be reflected in opposite changes in airway wall dynamics [23]. The lung microsections technique obviates surface tension (by eliminating the air-liquid interface), the transmural pressure gradient, allowing detailed studies of the diametrically opposing actions of the ASM per se, and its parenchymal tethering attachments [12]. In a studies of a murine elastolytic model of emphysema, we were therefore surprised to find airways in which parenchymal attachments had been substantially disrupted could nonetheless exhibit airway reopening upon wash-out. This suggests that there is some structure or force resident within the airway wall *per se* which also contributes to maintaining airway patency [38, 39].

### Videomicrometry of lung microsections

**Fig. 1:**
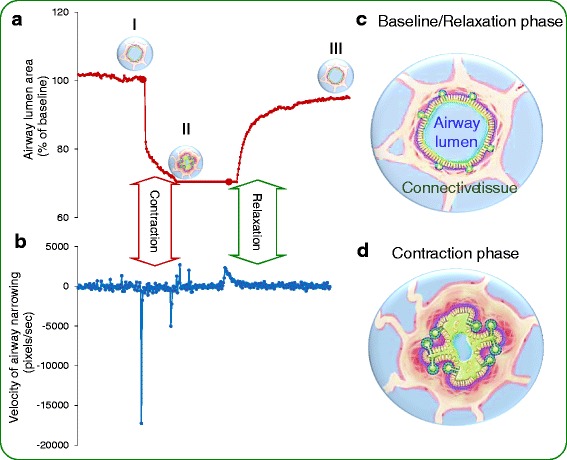
**a** Airway diameter (measured at 10 second intervals, during different phases (I, II & III) of airway contraction and relaxation, and each phase represent airway at baseline (I), contracted (II) and partially relaxed (III) respectively. **b** Moment-by-moment velocity of changes in airway diameter. **c**, **d** Cartoons showing airway movements during the phase of contraction and relaxation

Lung microsections provide a valuable source to assess interaction of ASM to its surrounding parenchyma (tethering) to different agonist in preclinical models of emphysema and asthma conditions [16, 39, 50]. Mice treated with a variety of noxious stimuli (elastase, cigarette smoke, over expression of TGF-β) in order to reproduce various aspects of COPD/emphysema result in various degree of tissue destruction and emphysematous changes [27, 70]. Lung microsections can be prepared through the agar inflated mice lungs, and cut in to slices ≈ 120 μm thick, then mounted on to a CCD-equipped microscope to visualize individual airways and matrix architecture during airway response to various stimuli through phase contrast microscope equipped with a high resolution CCD solid state video camera (CV-252 Nikon, Japan), and recorded in time lapse (3-ms exposure, 0.2 frames/sec) using image acquisition software (Video Savant, IO Industries, London, ON, Canada). Video images will be further analyzed using Scion image analysis software (Scion, Frederick, MD): the 10-bit video images were converted to binary, then cross-sectional area of the airway in each frame was measured by pixel summing. The velocities of this constrictor response to challenge with acetylcholine (Ach), as well as the relaxant response to washing off of Ach, were both obtained by taking the first derivatives of the changes in airway diameter with respect to time [16, 38, 39]. It has been reported in various preclinical settings that challenge of the airway tissues with neurotransmitters (10^−5^ to 10^−8^ Acetylcholine, 10^−5^ 5- hydroxytryptamine or 5-HT) and/or relaxants (10^−6^ M Isoproterenol) leads to changes in diameter of airways; these responses can be easily videotaped and analyzed using lung microsection techniques for magnitudes and velocities of contraction, both of which reflect changes in the interactions of the smooth muscle with the surrounding tissue [2, 39, 44]. In addition to this, various targets can be tested to assess the roles of inflammation, fibrosis, metalloproteinases and other parameters in these structural/functional changes [16]. After subjecting the tissues to a wide range of physiological and pharmacological manipulations in lung microsections, the tissues can be later used for other studies, which include wide range of applications including histochemical studies to assess the degree of inflammation (immune cellularity; levels of molecular markers), amounts of connective tissue proteins and the molecular agents, which regulate them [39]. Lung microsections approach is not affected by most of the physical parameters used during processing and recording microsections, which include concentration/volume of agar used during inflating the lungs [39]. However, it is affected by the age, strain, airway architecture, concentration and types of inducers used for airway response. Others have used 0.75 %–1 % agarose to inflate rodent lungs prior to cutting into slices 500-1000 μm thick [18], [7] or 250 μm thick [50]. This approach was then modified using 2 % agarose to inflate and stiffen the lungs prior to cutting into slices approximately 100 μm thick [9]. [4] Adler et al. have previously demonstrated that 2 % agarose-filled lungs have a shear modulus similar to that of air-filled lungs [1]. In addition, Influence of video recording rate/rate of data acquisition (video frame rate 10fps-1fps-0.5fps) also influence the estimation of velocities of airway narrowing (referred to as aliasing) because data acquisition at higher rate (10fps) produce the substantial amount of “noise” due to minor twitches in the muscle, drifting debris and subtle changes in illumination and this “noise” hampers any accurate determination of peak velocity of airway closure (Fig. 1).

### Data analysis and interpretations

The experimental protocol to assess airway responsiveness to cholinergic stimulation is illustrated in Fig. 1. To plot an airway response, microsections can be challenged with neurotransmitter, acetylcholine (Ach) to obtain a maximal constrictor response. This elicited a rapid and dramatic decrease in airway diameter that reached a peak within 5 min (Fig. 1a). Later, effects of neurotransmitter can be neutralized with washing from the tissues by perfusing with regular HBSS for 5–10 min, during which time the airway relaxed back toward its baseline diameter (Fig. 1a). The first order derivative of this contraction trace will yield moment-by-moment velocities of contraction and relaxation (Fig. 1b); the mean peak velocity of contraction (V_con_ in pixels/sec) is always significantly faster than the mean peak rate of relaxation (V_rel_; in pixel/sec) [38, 39]. It has previously been shown that the magnitude of airway narrowing measured in murine lung slices can vary with airway size over a very wide range of airway sizes [5]. Airway areas and velocities of narrowing were measured in airways from the right cardiac lobe; the animals used in this study were all from the same batch in order to control for other influences such as age, handling, etc. [38, 39] In addition, this technique also validated how the rate of video recording might impact the measurement of airway movements, to minimize the size of the video files produced: a 45 min video recorded at standard video rates constitutes 162,000 images, resulting in a file size measured in several hundred megabytes for a single recording [38, 39]. On the other hand, given that the bulk of the constrictor response occurs within the first 10 seconds following stimulation, a video rate which is too slow will not adequately capture the peak instantaneous velocities of the mechanical responses [16, 39]. Using several different videos of a single common bronchoconstrictor event, each captured using time-lapse videography with different rates, we have reported that capture rates of 0.5–1 Hz (one frame every 1–2 seconds) is sufficient to not only determine the latency and magnitude of the peak velocity, but also to reveal more subliminal mechanical events such as oscillations in constriction velocity at the onset of cholinergic contraction. The latter likely reflect modulation of the contractile apparatus as it begins to develop mechanical force [38, 39].

### Applications

Videomicrometry of lung microsections has been used extensively to study a wide variety of mechanical responses and key information pertaining to the connective tissue matrix is likely present in the time-course of airway narrowing/opening [17, 18, 40, 50]. Earlier studies have by and large examined only the absolute magnitudes of airway narrowing, paying little or no attention to the dynamics of those changes [63]. This review basically highlights the significance of lung microsection technique to investigate the instantaneous velocities of narrowing, allowing us to not only derive the latency and magnitude of the peak velocity, but also to scrutinize smaller events such as minor oscillations of constriction velocity at the onset of the cholinergic response [38, 39]. As such, this approach could prove to be invaluable in studying novel aspects of excitation-contraction coupling in ASM, as well as the interactions between the ASM and its surrounding parenchymal matrix [38, 39]. Duguet et al. have reported that airway constriction and velocity of narrowing is governed by the elastic load on the airway wall [22]. This techniques has been validated and utilized extensively both in preclinical models [38] and in investigating clinical human samples [57, 58, 71]. This techniques is precisely sensitive, and measurements are not affected the accuracy of the airway-ECM movements to a number of experimental factors, including those pertaining to the tissue *per se* (age and strain of the animal; size of the airway), to the preparation (concentration of the agarose; degree of inflation), and the rate of video acquisition [38], instead only affects the magnitude and velocity of responses in airway-ECM interactions, which may differ in different pathological conditions [38, 39]. Finally, given that others have documented variations in the magnitude of the airway narrowing response over a very wide range of airway sizes [9, 41, 52], this technique is also unaffected with airway size: in fact, the velocities of bronchoconstriction in large and small airways have no significant difference on dynamics, although we should point out that we used a smaller range of airway size than was the case in the other study [5]. This technique and related measurements are also independent of other physical parameters used in inflating medium (agarose) concentration, used to stiffen the lungs for slicing [38, 39]. Although, it seems that higher concentrations of agarose within the parenchymal spaces would act as a greater impedance to stretching of the tissues and thus to airway narrowing. On the other hand, greater degrees of lung inflation might be expected to influence the magnitudes of airway narrowing [33, 36, 51], and perhaps also the rates of constriction/relaxation, although concentration of inflating medium have no substantial dependence upon the concentration of agarose used, nor upon the degree of inflation [38, 39]. In addition to investigate lung pathology in detail including ECM distribution and pattern, it may provide useful in studies of various physiological aspects of the contractile apparatus itself. [38, 46] Thin microsections are superior to standard tracheal strips and bronchial rings in that there is less diffusional barrier between the bathing medium (containing exogenously applied drugs) and the smooth muscle layer: this likely account for the substantially longer latency for peak constriction in tissue strips *versus* lung slices [18, 38–40, 50]. Finally, contractions recorded in those latter preparations are usually obtained under isometric conditions, and occasionally under isotonic conditions, whereas constrictions recorded in lung microsections are auxotonic in nature (as is the case in vivo) [9–11]. As demonstration of the ability of this technique to resolve changes/differences in physiological events and in addition It may even be possible to use this approach to address molecular questions related to the contractile apparatus, such as cross-bridge cycling or the “series-to-parallel transition” which occurs during the first 30 seconds of contraction; tracheal strips and bronchial rings are not suited to this purpose due to their higher tissue mass and inertia [16, 38, 46, 50]. The technique which I discuss here is therefore ideal for investigating the impact of tethering forces on airway mechanical responses: it goes without saying that such interactions are precluded in tracheal strips and bronchial rings (which lack the parenchyma), in addition, structurally, the lung tissue is a viscoelastic material, and, viscoelastic materials display hysteresis [25]. As reported in major clinical pathology of emphysema, the elastin fibers are degraded: as such, the airway wall does not recoil so strongly [27, 39, 71]. On the other hand, pulmonary fibrosis with aging or in disease would be reflected in opposite changes in airway wall dynamics [23]. Increased ASM mass, seen in asthma and various animal models of asthma, is expected to manifest as changes in not only the magnitudes of airway narrowing (measured using existing techniques) but also in the rates of narrowing (not available through current approaches). Thus, it would be useful to apply this technique to studies of emphysema, fibrosis, lung development, airway hyperresponsiveness [2, 16, 39, 46]. It can be applied both to animal lung preparations as well as to the small samples of human tissues, which are frequently resected in the operating suite [71].

In addition to using this approach to study lung pathology, it may prove useful in studies of various physiological aspects of the contractile apparatus itself. [38] Finally, contractions recorded in those latter preparations are usually obtained under isometric conditions, and occasionally under isotonic conditions, whereas constrictions recorded in lung slices are auxotonic in nature (as is the case in vivo) [38, 39]. Lung microsection technique is a useful tool that play a key role in understanding lung physiology in health and disease in major pulmonary diseases including asthma, COPDs, but it has some limitations. Many features of the thin lung slice that make it suitable for the study of multicellular activity but not suitable with isolated single cells. Anatomically, lung slice is not suitable for drug delivery experiments due to epithelial barrier is circumvented at the cut edge of the slice, which make it difficult to mimic drug deposition in the airway lumen that is a common therapeutic approach. In addition, many studies are conducted with static tissues or cells and with respect to lungs anatomy this is very important in view of the repetitive cycling of contraction and relaxation during respiratory cycle. In asthma condition, airway smooth muscle stiffness is thought to be increased as result of a loss of response to this movement but this condition cannot be translated in lung slice [46, 61]. Consequently, it is worth developing approaches to create a “breathing” lung slice.

Video-micrometry of lung microsections has been used extensively to study a wide variety of mechanical responses [38, 39] (R). The lung microsections approach may also be useful for studies of lung dynamics and associated structural deformities in airway compartments during disease and against various inducers [9, 16, 39, 50]. In this review, we highlighted the clinical benefits of lung slice technique, which employ to investigate estimations of instantaneous velocities of narrowing, allowing us to not only derive the latency and magnitude of the peak velocity, but also to scrutinize smaller events such as minor oscillations of constriction velocity at the onset of the cholinergic response [16].

## Conclusions

In conclusion, measurements of airway constriction velocities should prove useful for studies of lung development, fibrosis, emphysema, allergen-induced airway hyper-reactivity, and basic physiology [46]. As such, this approach could prove to be invaluable in studying novel aspects of excitation-contraction coupling in ASM, as well as the interactions between the ASM and its surrounding parenchymal matrix [16, 39, 46]. It can be applied both to animal lung preparations as well as the small samples of human tissues which are frequently resected in the operating suite [71].
